# Family caregivers' experiences of caring for neonates undergoing enterostomy in China: A qualitative study

**DOI:** 10.1002/nop2.1350

**Published:** 2022-08-30

**Authors:** Zhi Hong Ni, Sheng Ding, Jin Hua Wu, Fang Wang

**Affiliations:** ^1^ Children's Hospital of Soochow University Suzhou China

## Abstract

**Aim:**

To describe the nursing experiences and care needs of family caregivers caring for neonates with enterostomies in China.

**Design:**

A qualitative study.

**Methods:**

We conducted semi‐structured interviews with family caregivers who care for neonates undergoing enterostomy. Family caregivers were selected using a purposive sampling method from three children's hospitals until no new data were generated (*N* = 26). Data analysis applied the thematic analysis method. The caregivers' experiences were described using qualitative content analysis.

**Results:**

The experience of family caregivers was described as growing in adaptation, where five main themes emerged: (1) complex emotional responses; (2) uncertainty about everything; (3) lack of confidence, anxiety and helplessness; (4) eagerness for professional support; (5) active adaptation, gain‐harvest and gain‐growth. These findings increased understanding and added knowledge on this topic that is rarely studied in China. Healthcare authorities and professionals should recognize and understand the lives and situations of family caregivers (whose neonates undergo enterostomy) to better identify their difficulties and needs.

## 1 INTRODUCTION

Neonate enterostomy is one of the common surgical methods for acute and severe neonatal intestinal diseases (Chong et al., 2019). It refers to the surgical formation of an opening into the intestine through the abdominal wall as a temporary artificial anus and is used to rescue the acute abdomen caused by neonatal necrotizing enterocolitis, intestinal perforation, intestinal necrosis, intestinal atresia, poor intestinal rotation, intestinal volvulus and anal atresia (Brunette, 2017). More than 100,000 neonates undergo enterostomy every year in China, and the incidence gradually increases (Li et al., 2019). Temporary stoma was left in the abdominal cavity after enterostomy; when the infants' intestinal function returns to normal, suture of the stoma is generally performed after 5–6 months (Miller et al., 2017). Infants have tender skin and poor resistance to infection. The incidence of various infections and complications after enterostomy is approximately 28%–70% (Li et al., 2019), and peripheral skin complications are the most common, being more common in extremely preterm neonates or those with extremely low birth weight (<1,000 g) (Bethell et al., 2017).

Neonates with postoperative enterostomy will enter the neonatal intensive care unit (NICU) for treatment and nursing by healthcare professionals for a period of time.

Compared with their peers, neonates undergoing enterostomy require more care after discharge, which can be challenging and causes considerable stress for parents and other caregivers (Msall, 2019).

Family‐centred care (FCC) is a proposed method to support family involvement in a child's care and reduce the stress associated with a child's critical illness by improving communication, supporting coping and reducing conflict (Coats et al., 2018). Modifications to support FCC include changes to the physical environment and modifications to policies and clinical interactions. Parents were only allowed to visit their child briefly in the NICU because of concerns about infection control. However, research has shown that infants were less stressed and more comfortable when their parents were present at the bedside (Gómez‐Cantarino et al., 2020). To help caregivers reduce anxiety and improve their skills in caring for newborns who underwent enterostomy, the NICU of children's hospital implements FCC allows longer family visits and changes the physical environment by moving from open floor plans to single rooms.

Parents of neonates who undergo enterostomy often experience more severe negative emotional reactions and psychological pressures than those experienced by parents of healthy newborns; their parents will continue to care for the enterostomy after the neonates are discharged from the hospital. Parents are not fully prepared for their role as enterostomy caregivers and even have difficulties in adapting to the role (Petteys & Adoumie, 2018; Sancar et al., 2020).

The difficult role of caregivers will directly affect the quality of neonatal care. Parents of neonates undergoing enterostomy often lack experience in providing stoma care and lack relevant medical knowledge, which leads to difficulties in caring for such infants (Spittle et al., 2018). Poor care often leads to various complications of stoma, affects the recovery of children's disease, prolongs the time of stoma closure surgery and even endangers children's life or affects their growth and development (Gao et al., 2021), neonates require ongoing care and support (Amorim et al., 2018; O’Donovan & Nixon, 2019).

Parents of neonates undergoing enterostomy have different concerns as they were transferred from the NICU to home (Garfield et al., 2016; Li et al., 2019). Many of these concerns can be addressed with improved discharge information exchange and anticipatory guidance. Supporting parents during this stressful and often difficult transition may reduce family stress, improve care and better outcomes for the infant.

This study aimed to understand the real experiences of family caregivers in caring for neonates undergoing enterostomy to construct a neonate undergoing enterostomy nursing mode for family caregivers.

## 2 METHOD

### 2.1 Ethics statement

This study was conducted following the Declaration of Helsinki (WMA, 1964). Research Ethics Committee approval was granted by the ethics committee of the Childrens Hospital of Soochow University (No 2021ks001). Informed consent was obtained from all participants. Caregivers could decline to answer specific questions and were free to ask for a break or terminate the interview at any point. Only the interviewer knew the identity of the participants, while the other researchers worked with anonymous data transcripts. All data were stored on a password‐protected hard drive that were used only for this project. In addition, no one outside the research group had access to the data. Participants who wished to obtain the results were informed that they could do so later, but only as aggregated data.

### 2.2 Study design

This study used a descriptive qualitative research design through semi‐structured interviews on family caregivers' experiences of caring for neonates undergoing enterostomy (Speziale et al., 2011). Methods have been reported here according to the Consolidated criteria for Reporting Qualitative studies guidelines (Tong et al., 2007).

### 2.3 Participants

This study had a qualitative, descriptive design and used semi‐structured, open‐ended interviews to analyse the caregiving experiences and nursing needs of family caregivers caring for newborns with enterostomies.

All interviews were conducted between January–November 2021 at the NICU in three children's hospitals in Jiangsu province, China. Purposive sampling was used to enrol neonates undergoing enterostomy who were in the NICU.

The neonates' inclusion criteria were as follows: (1) neonates who underwent enterostomy for the first time; (2) age < 28 days. The exclusion criteria were as follows: complicated with other serious diseases, congenital heart disease, malignant tumour, genetic metabolic disease, etc. They were selected purposively from the NICU in three children's hospitals in Jiangsu province between January–November 2021.

Caregivers' inclusion criteria were as follows: (1) caregivers' age ≥ 18 years old; (2) as the primary caregiver; (3) have normal communication and expression skills. Exclusion criteria for caregivers were as follows: (1) caregivers have a mental illness; (2) experienced major stress events within 3 months (bereavement, divorce, etc.). After applying the selection criteria, a total of 26 caregivers (16mothers and 10 fathers) aged 18–38 years were enrolled in this study. Furthermore, 14 male neonates and 12 female neonates were included in the study. The general characteristics of the neonates and their caregivers are listed in Tables 1 and 2, respectively.

**TABLE 1:** Demographic data of the newborns

Variable	*N*	*F* (%)
Gender		
Male	14	61.5
Female	12	38.5
Birth weight (g)		
<2000	5	19.2
2001–2,500	13	50.0
>2,500	8	30.8
Diagnosis		
Neonatal necrotizing enterocolitis	11	42.3
Intestinal necrosis	6	23.1
Intestinal atresia	5	19.2
Anal atresia	4	15.4

**TABLE 2:** Demographic data of the caregivers

Variable	*N*	*F* (%)
Education		
Middle school	5	19.2
Junior college	10	38.5
University	11	42.3
Age		
<25	6	23.1
26–30	9	34.6
>31	11	42.3
Occupation		
Unemployed	4	15.4
Company worker	8	30.8
Agricultural worker (poor)	5	19.2
Office clerk	9	34.6
Residence		
City	11	42.3
County	15	57.7
Caregiver		
Mother	16	61.5
Father	10	38.5

### 2.4 Data collection

Participants were briefed about the study and informed of their right to withdraw at any point. Face‐to‐face interviews were conducted and audio‐recorded in the participants' private hospital offices at a time convenient to them. Written informed consent was obtained at the beginning of the interviews.

A senior researcher (NZH) performed the interviews and trained less experienced colleagues. NZH is an experienced nurse with a PhD. All researchers experienced in conducting qualitative research.

An interview guide was developed and confirmed by the authors and was pilot‐tested in the first three interviews, which resulted in minor revisions. These three interviews provided relevant information; therefore, they were included in the data analysis.

Each interview started with broad questions: (1) What did the caregivers already know about enterostomy? (2) Did the health professional tell the caregivers everything they needed to know, and did they want to know anything else? (3) How did caregivers feel during the care process? (4) What was the most challenging problem encountered during the care process? (5) How did they solve the difficulties encountered? All interviews were conducted in Chinese and were digitally recorded and transcribed by multiple research assistants fluent in Chinese. The recordings were transcribed verbatim within 24 h. Interviewing skills, such as active listening and open‐ended questions, were used. Furthermore, non‐verbal information, such as obvious pauses, sobbing, and other speech features, was also recorded in the transcript.

Sampling also considered the need to maximize diversity. Recruitment continued until the data were saturated. If no new information was obtained, the data were saturated after 26 interviews. Each interview lasted 30–40 min. Three additional interviews were conducted to confirm data saturation. In the last three interviews, data analysis led to the emergence of repetitive codes and no new code was obtained. In addition, the decision to reach data saturation was made by reviewing the codes and categories by the research team members and two experts who were not part of the research team. (FitzGerald et al., 2008).

### 2.5 Data analysis

This study applied the thematic analysis (Kao, 2001). First, the given transcript was read several times to determine any hidden significance. Second, the key points, events or feelings in the verbatim transcripts were reviewed and were given a preliminary open coding. Preliminary categorization of the open coding was conducted in order to discover the conceptual attributes, and those conceptual attributes were then named with appropriate terms according to the initial categorization. Third, the related names were compared and summarized into sub‐themes. The common sub‐themes between the transcripts were then summarized to form themes. The first author coded the data and verified it with the third author to refine the different concept categories and identify relationships among these categories. Reporting adheres to the consolidated criteria for reporting qualitative research checklist. Data analysis was performed using NVIVO software (QST International, Cambridge, MA, USA).

### 2.6 Rigour

In this study, to assure the trustworthiness of the data, criteria including credibility, dependability, transferability and confirmability, as proposed by Lincoln et al. (1985), were used. To ensure the credibility of the data, the researcher had a long‐term relationship with the participants, which aided trust and openness. Dependability was determined through review of data and coding by a co‐worker. Two external examiners with Ph.D. in nursing who had experience in qualitative research were asked to review the interview transcripts, the initial coding and the categories. Any disagreements were discussed in a meeting and consensus was achieved among the research team about the correct coding. Regarding transferability, the characteristics of the research population and the research process were described clearly and accurately, to make it possible to follow the research path and key decisions made in the analysis. The confirmability of the data was established by the researchers actively putting aside their thoughts and assumptions about the topic, accurate recording of the research procedure and documentation, refraining from the deep review of texts. This was all assisted by input from the rest of the research team.

## 3 RESULTS

Through data analysis, we identified the following five themes: (1) complex emotional response; (2) uncertainty about everything; (3) lack of confidence, anxiety, and helplessness; (4) eagerness for professional support; (5) active adaptation, gain‐harvest and gain‐growth. (Table 3). Each theme is described below with supporting quotes from the participants.

**TABLE 3:** Superordinate and sub‐themes identified in the analysis

Themes	Sub‐themes	Categories
(1) Complex emotional response	Panic and worry	Parents had to go through a series of processes transfer to NICU, parents felt panic and worry.
		The unknown and severity of the disease made parents panic and worry
	Guilt and remorse	Parents blamed themselves and doubted whether they were competent for the role of parents.
		Parents felt a deep sense of self‐blame and guilt after the child's birth
(2) Uncertainty about everything	Uncertainty of disease prognosis	Complications in the temporary stoma of the abdomen after the operation, which made parents feel uncertain about the prognosis of the child.
		Parents had a sense of uncertainty about their child's future because they were worried about the child's condition.
	Uncertain about the child's current situation	Parents lacked relevant knowledge about the progression of the disease, and they hoped to receive professional explanations from healthcare professionals to alleviate their anxiety.
		Parents could not accompany their children. They hoped to know the situation of the children at all times
(3) Lack of confidence, anxiety, and helplessness	Lack of care confidence	Parents doubted that they were competent for the role of caregiver.
		Parents worried about their ability to take care of their children.
	Anxiety and helplessness come from multiple role conflict	Parents worried about their ability to take care of the baby after returning home.
		Parents felt pressure about their ability to take care of the baby after returning to work.
(4) Eagerness for professional support	Lack of skill and eagerness for professional support	Due to care pressure, parents were eager to receive diversified and continuous professional support.
		Parents were not fully qualified for the role of caregiver, and they hoped the medical staff can come to help.
	The heavy burden of care	The care of neonates with enterostomy requires more energy and financial resources, bringing heavy care and economic burden on parents and the whole family.
		The caregiver's family pays a lot of money for the child's treatment, but is physically and mentally exhausted.
	High social pressure and intentional concealment of the disease	Parents refused any visitors.
		Parents did not communicate with others and concealed the child's condition.
(5) Active adaptation, gain‐harvest, and gain‐growth	Actively adapt to the role of caregiver	Parents learned relevant medical knowledge to provide better care for children.
		Parents actively took care of the child.
	Harvest growth experience	Parents gained self‐growth by taking care of children.
		Parents became more and more responsible.

### 3.1 Complex emotional response

#### 3.1.1 Panic and worry

During this period, parents usually have to go through a series of processes from ambulance transfer, emergency surgery and transfer to NICU. All the while, the parents feel a sense of panic and extreme anxiety.

The doctor said that my child had intestinal perforation and needed surgery. I was very worried and panicked because the child was only 6 days old and undergoing surgery at such a young age. Caregiver #1.

I felt very anxious at that time; I was worried whether the child could stand the operation. Caregiver #2.

The lack of knowledge about the disease and the severity of the disease cause parents to panic and worry, and it is difficult for parents to adjust to the role of caregiver.

The doctor said that my baby's disease was very serious and if it gets worse, my child will die; I am scared, I did not know what to do. Caregiver #6.

At that time, I felt that my baby's illness was very serious, and I was very flustered. I wanted to save my baby's life. The child must survive. Caregiver #22

#### 3.1.2 Guilt and remorse

At the initial stage of hospitalization, due to insufficient understanding of the disease, parents often blame themselves, even doubt whether they are competent for the role of parents.

Our twins are test‐tube babies. I think it may be my poor health that my babies suffer as soon as they are born. Caregiver #20.

I am not a qualified mother. I had a bad appetite; I ate less, and I was often in a bad mood when I was pregnant; I think it caused my child to be born prematurely and with a weak physique? Caregiver #16.

The child was diagnosed with a congenital malformation after birth, and the parents felt deeply guilty and at fault.

When the doctor told me that the child had a congenital imperforate anus, I thought I must have done something immoral in my last life, and this retribution is on my child. Caregiver #18.

### 3.2 Uncertainty about everything

#### 3.2.1 Uncertainty of disease prognosis

After neonates undergo enterostomy, the patient's condition has been alleviated, but there may be a series of complications in the temporary stoma of the abdomen after the operation, which often makes parents feel uncertain about the prognosis of the child.

The doctor told me that the operation was successful, and my mood improved immediately at once, but he said that there were many complications. Now I am still worried about whether my child will get better. Caregiver #8.

I do not know if the child's stool will be normal. What should I do in case of faecal incontinence? I am worried that it will affect my children for a lifetime. Caregiver #17.

Parents experience a sense of worry and uncertainty about their child's future, given the seriousness of the condition.

The doctor said that a large section of the baby's intestines was broken and surgically removed. Will the baby's digestive function be affected? Caregiver #9.

The child will undergo a second operation in a few months. Can such a young child stand it, and there will be a long time in the process. The doctor said that there would be many uncertain factors. I do not know how much pain the child will experience. Caregiver #15.

#### 3.2.2 Uncertain about the child's current situation

After an enterostomy operation, neonates are often transferred to the NICU for further treatment. Parents lack relevant knowledge about the progression of the disease, and they hope to receive professional explanations from healthcare professionals to alleviate their anxiety.

The doctor made a hole in the child's belly and stuck a bag to collect stools. How long will the bag be stuck, and how can I change it if it is dirty? We do not understand. The healthcare professionals must be professional in this regard, so I hope they can tell us more. Caregiver #2.

What does the stoma on the baby's stomach look like? Will the baby feel pain? Now I hope to know the child's condition at any time. Caregiver #13.

Parents are not permitted to accompany their children. They are anxious to know about their child's condition at all times and be able to see their them.

The baby is treated in the NICU. I cannot see the child. I am very worried. I hope healthcare professionals can take more photos or videos of the child so that our whole family can have a look and my family's anxiety can be relieved a little. Caregiver #19.

The baby has suffered so much; I cannot accompany him. I feel so sorry for him. I hope I can go in and see and hug him, let him know that his mother is always there. Caregiver #5

### 3.3 Lack of confidence, anxiety and helplessness

#### 3.3.1 Lack of care confidence

Neonates with enterostomy generally need stoma closure surgery in about 6 months; parents should perform the nursing work of enterostomy for a long time after discharge. At this stage, with the approach to discharge, parents doubt that they are competent for the role of caregiver.

We are confused as to how to do it when we get home. The nurse has told us clearly, but we are still worried when we go back to take care of the baby because it is too difficult for us. Caregiver #12.

We are really afraid to go come home with that stoma bag attached to the baby. If it does not operate correctly, will the child be infected? Caregiver #14.

Parents have no experience in taking care of children and therefore doubt their parenting skills.

This is our first child. We do not have care experience. Now we must be in a hurry to face this enterostomy care. Caregiver #3.

#### 3.3.2 Anxiety and helplessness come from multiple role conflict

Parents began to worry and feel pressure about their ability to take care of the baby after returning home and returning to work.

After the baby is discharged from the hospital, I need to get back to work. Only my mother and my wife take care of the baby. The baby problem is quite difficult, and I am afraid they cannot handle it. Caregiver #26.

After the baby comes home, we have to do many care tasks, but my mother‐in‐law has no knowledge and does not understand anything. My husband has to go to work and cannot ask for leave anymore. After giving birth to a child, I am in poor health and feel that I do not have enough energy, and I am so tired! —Caregiver #24.

### 3.4 Eagerness for professional support

#### 3.4.1 Lack of skill and eagerness for professional support

Home care for children after discharge requires parents to master highly professional nursing skills, such as replacement of the stoma bag, which is a great challenge for parents. Due to care pressure, parents were eager to receive diversified and continuous professional support.

Replacing stoma bags requires exquisite technology. When I first changed the stoma bag, it took a long time and the bag still did not stick well. I hope someone could guide me. Caregiver #4.

It is really difficult. At one time, I changed the stoma bag, but the order of stoma powder and skin protective film was reversed. The bag could not stick. The baby cried loudly, and I felt so stupid. Caregiver #7.

Parents are not fully qualified for the role of caregiver and hope that medical staff would offer assistance.

At one time, there was blood in the baby stoma, and I was so scared. I hope someone could give me more guidance, even by telephone and video. Caregiver #10.

I really hope that healthcare professionals can do a home visit to supervise what our families have done so far and how the children are recovering and also give me some guidance. Caregiver #21.

#### 3.4.2 The heavy burden of care

The care of neonates with enterostomy requires more energy and financial resources than ordinary children, bringing a heavy care and economic burden on parents and the whole family.

My child's surgery cost a lot of money, and this stoma bag is not cheap. I think the financial burden is heavy. Caregiver #23.

I do everything for the baby myself. I am really tired, but I feel indebted to him. I just hope he that he gets better soon. Caregiver #25.

The parents pay for the child's expensive treatment and are physically and mentally exhausted.

My husband works in another city. My mother‐in‐law is old. In addition to the baby, there are many more things. No one can help me, I am really tired, I also do not want my husband to come back to work, but we need money. My child needs a lot of money for future treatment. Caregiver #11

#### 3.4.3 High social pressure and intentional concealment of the disease

Due to the child's disease, the parents refused any visitors (relatives and friends); they did not want to tell others about their child's disease.

I told my relatives that the children had health problems and their immunity was low. I asked them not to come home as much as possible. Caregiver #3

I dare not invite my friends to my house. They may see the bag on the baby's stomach; I am afraid others will know about the child's illness. Caregiver #1.

Parents isolate themselves, by avoiding social contact, in an attempt to conceal the child's condition.

My child has not been out since he came home. We are worried that others will know about the child's illness. Caregiver #7

### 3.5 Active adaptation, gain‐harvest, and gain‐growth

#### 3.5.1 Actively adapt to the role of caregiver

At this stage, parents actively adjust their mentality, gradually adapt to the role of caregivers and actively find and learn relevant medical knowledge, hoping to provide better care for children.

I gradually gathered information from some professional information on the nursing knowledge website and learned how to take care of the baby. Caregiver #23.

At the beginning, I was not good at handling the stoma bag. I went to the nursing clinic of the hospital to learn about it. Now I have a very good technique to change the bag. Caregiver #2.

#### 3.5.2 Actively adapt to the role of caregiver

At this stage, parents actively adjust their mentality, gradually adapt to the role of caregivers and actively find and learn relevant medical knowledge, hoping to provide better care for children.

I gradually gathered information from some professional information on the nursing knowledge website and learned how to take care of the baby. Caregiver #23.

At the beginning, I was not good at handling the stoma bag. I went to the nursing clinic of the hospital to learn about it. Now I have a very good technique to change the bag. Caregiver #2.

The parents are actively caring for the child to undergo stoma closure surgery in a few months.

I cannot be hurried when taking care of the child, The child will have stoma closure surgery after a few months. Caregiver #15.

#### 3.5.3 Harvest growth experience

Parents have gradually changed from anxiety, tension and lack of confidence to a positive face and gratitude. Many people feel that they have gained self‐growth by taking care of children.

I am the only child in my family. I do not need to worry about it since I was a child. This time, I feel that I have experienced great ups and downs in my life. My baby has suffered a lot since birth. My parents are old and lack energy. I have to take on family responsibilities. Caregiver #18.

I do not know how difficult it is to be a parent before I have children, but I really think it is not easy to raise a child when I start taking care of my baby. When I think of my parents' efforts, I really need to be grateful for them. Caregiver #8.

While gaining experience in caring for their children, parents become increasingly responsible.

After this experience, our family became more united. I have become more and more responsible. My knowledge and skills in care are also increasing. I must make my child grow up healthy. Caregiver #16.

## 4 DISCUSSION

This study explored the experiences of family caregivers in China who care for neonates undergoing enterostomy. The results showed that the parents of neonates developed complex emotional responses before the neonates underwent enterostomy, which is consistent with the results of other studies (Li et al., 2019; Miller et al., 2017). In the traditional Chinese idea, it is important to have a healthy child in the family (Li & Freedman, 2020). In the diagnosis period, when neonates with enterostomy are admitted to the hospital, they often need urgent surgical treatment due to their serious and life‐threatening condition, and their families often fall into panic.

During the postoperative period, the children entered the NICU for further observation. At this stage, the NICU is in a closed management mode, and family members are forced to separate from the neonates, resulting in a series of negative emotions, such as separation anxiety and reactions to psychological stress to varying degrees (Heo & Oh, 2019). The diagnosis of children's diseases, changes in their condition after surgery and recovery after surgery will affect their parents' emotions. During the hospitalization of children, healthcare professionals must provide timely and objective answers to parents on the progress of the children's condition so that the parents can obtain the encouragement and strength offered by healthcare professionals and improve their coping abilities. The study found that parents often voluntarily undertake the care of children. However, facing complex stoma nursing, they often appear anxious and uneasy, unable to judge whether their care is correct, consistent with a previous study by Kim et al (Kim et al., 2020). In addition, the mothers of newborns experienced varying degrees of self‐blame and guilt (Granero‐Molina et al., 2019). They believed that they were somehow responsible for their children's ill health. This finding is in line with the findings reported by Lundqvist et al. (Lundqvist et al., 2019) that showed that more than 90% of mothers of children discharged from the hospital feel uneasy in their parenting life.

Almost all parents in this study could not tolerate a long separation from their children and hoped that visiting times would be extended or the visitation system would be changed. It was another finding of this study. Although remote devices, such as visiting machines, have been installed in the NICU, parents were not satisfied with seeing only videos or images of their children without sound. They hoped to see the treatment of their children in a more intimate manner and communicate with health professional face‐to‐face (Villamizar‐Carvajal et al., 2018).

This study found that in the postdischarge period, parents often feel that their stoma care ability is insufficient and lack of confidence in caring for their children. Although neonatal enterostomy is mostly temporary, the stoma will be closed after 6 months. Parents must learn stoma care skills after discharge. Studies have confirmed that parents' stoma care ability is inversely proportional to the incidence of children's stoma complications (Christian et al., 2018). Good care ability is an important factor in reducing stoma complications, changing children's health outcomes and promoting their growth and development (Gad et al., 2017). Therefore, healthcare professionals should pay attention to improving the parents' care ability of stoma. To date, knowledge and skill guidance for parents of children with enterostomy is limited to communication during hospitalization and follow‐up after discharge. This study found that parents had a strong desire to obtain enterostomy knowledge and skill guidance since the child was discharged; parents also hope that the healthcare professional evaluates whether their care behaviour is correct and hope the forms of stoma care teaching diversify, obtain continuous and professional care guidance (Liu et al., 2018). Therefore, healthcare professionals should formulate corresponding guidance plans for different stages and implement stoma knowledge and skills guidance to help parents adopt the role of caregiver. After the child is discharged from the hospital, medical staff should make full use of the network platform, such as establishing a WeChat group to promote nursing knowledge, uploading nursing videos for parents to watch and learn, such as how to replace stoma bags with more difficult nursing (Liu et al., 2018). At the same time, health professionals provide guidance and evaluation to caregivers in combination with a stoma specialist clinic and family visits, providing diversified forms of education, providing guidance on enterostomy knowledge and skills, and improving the nursing ability of parents.

The benefit finding refers to the individual, social, psychological and spiritual benefits perceived by an individual experiencing stress or posttraumatic events (Fu et al., 2020). Studies have shown that when children suffer from serious diseases, their parents can grow rapidly and become mentally stronger (Cheng et al., 2017). To do this, we encouraged caregivers to reappraise challenging situations, whether real or hypothetical, in positive ways and as many ways as possible. Improved patience and mental strength helped them face other challenges in life. The evidence reviewed would suggest that psychological and social resources may ameliorate the negative impact of caregiving and even lead to positive affect, life satisfaction and benefit findings (Cassidy et al., 2014).

The study found that in addition to a series of negative feelings, parents also gained a positive growth experience in the process of taking care of children; they learned to be grateful and cherish life, had the courage to assume family responsibilities and had a closer family relationship. The sense of benefit can help caregivers reconstruct their cognition and improve their resilience to negative events to reduce caregivers' negative reactions and promote their physical and mental health (Cheng et al., 2021). Positive growth can also help caregivers establish confidence in overcoming diseases, improve the quality of care and improve the quality of life of children (Peacock, 2014); this is consistent with the results of our study. It can also help caregivers establish confidence in overcoming diseases, improve the quality of care and improve the quality of life of children (Tandberg et al., 2018), which is consistent with the results of this study. In the process of taking care of children, caregivers stimulated the sense of benefit from the disease, alleviated bad emotions, and improved the quality of parental care (Silveira et al., 2018; Spittle et al., 2016).

The nursing of enterostomy neonates involves many disciplines, such as neonatal surgery, stoma specialty, nutrition and psychology. The setting of best practice for enterostomy must be jointly determined by a multidisciplinary team with professional knowledge and health professional skills to ensure the nursing quality of stoma management. During the interview, it was found that parents faced many problems in the care process: how to feed, how to judge whether defecation was normal, how to correctly replace the stoma bag, how to adjust their mentality and reduce the sense of shame. They were eager to get the help of professionals in many aspects. Therefore, health professionals should play an active leadership role and build a multidisciplinary team continuous nursing plan, including discussing and formulating the stoma management plan and follow‐up plan with ostomy therapists, surgeons, nutritionists and psychological counsellors (Yu et al., 2020) to truly realize the continuity of nursing intervention. In addition, we should actively create a multidisciplinary joint clinic, strengthen the hospital community cooperation model, establish a perfect social support system and provide more high‐quality continuing care to parents of children with enterostomy.

## 5 IMPLICATIONS FOR PRACTICE

This study describes the care experiences and needs of caregivers' experiences of neonates undergoing enterostomy. The experiences of the caregivers of neonates undergoing enterostomy are complex. Hence, we need to sufficiently train healthcare professionals staff so that they identify the needs of caregivers and provide them with targeted intervention that meets their demands.

Healthcare professionals need to pay greater attention to the caregivers' experiences of neonates undergoing enterostomy, in order to provide a theoretical basis for constructing a nursing mode for neonates undergoing enterostomy and scientifically exploring the healthcare management system for neonates undergoing enterostomy.

## 6 LIMITATIONS

A potential limitation of the study is the small number of interviews performed. We interviewed the caregivers of neonates undergoing enterostomy in the diagnosis period, postoperative period and postdischarge period. In future studies, we will conduct a long‐term and sustained study of the caregivers of preterm infants. However, the interviews were rich in content and included a diverse study population in terms of cultural and social backgrounds. We acknowledge that minor aspects might not have been sufficiently covered or that other participants from different contexts may have differing views.

## 7 CONCLUSION

The complex emotional response, uncertainty about everything, lack of confidence, anxiety and helplessness, eagerness for professional support, active adaptation, gain‐harvest and gain‐growth resulted in caregivers growing in adaptation, describing a picture of family caregivers in China caring for neonates undergoing enterostomy.

Healthcare authorities and professionals should recognize and understand the lives and situations of family caregivers since their neonates undergo enterostomy to better identify their difficulties and needs. Appropriate and effective support, both from government and society, should be planned and implemented for family caregivers caring for neonates undergoing enterostomy and maintaining the quality of their own lives.

## AUTHOR CONTRIBUTIONS

All authors participated in the study design. NZH collected the clinical data, and data analysis was conducted by all the investigators. DS wrote and revised the draft and subsequent manuscripts. WJH and WF assisted with drafting and revising the manuscript. All authors read and approved the final manuscript.

All authors have agreed on the final version and meet at least one of the following criteria [recommended bythe ICMJE (http://www.icmje.org/recommendations/)]:

• substantial contributions to conception and design, acquisition of data or analysis and interpretation of data;

• drafting the article or revising it critically for important intellectual content.

## FUNDING INFORMATION

The authors thank the Children's Hospital of Soochow University key research project for funding this study number (2021ZDPY06).

## CONFLICT OF INTEREST

No conflicts of interest exist.

## Data Availability

The data that support the findings of this study are available from the corresponding author upon reasonable request.
